# Pathway-BasedFeature Selection Algorithm for Cancer Microarray Data

**DOI:** 10.1155/2009/532989

**Published:** 2010-03-03

**Authors:** Nirmalya Bandyopadhyay, Tamer Kahveci, Steve Goodison, Y. Sun, Sanjay Ranka

**Affiliations:** ^1^Computer and Information Science and Engineering, University of Florida, Gainesville, FL 32611, USA; ^2^Anderson Cancer Center Orlando, Cancer Research Institute Orlando, FL 32827, USA; ^3^Interdisciplinary Center for Biotechnology Research, University of Florida, Gainesville, FL 32611, USA

## Abstract

Classification of cancers based on gene expressions produces better accuracy when compared to that of the clinical markers. Feature selection improves the accuracy of these classification algorithms by reducing the chance of overfitting that happens due to large number of features. We develop a new feature selection method called *Biological Pathway-based Feature Selection (BPFS)* for microarray data. Unlike most of the existing methods, our method integrates signaling and gene regulatory pathways with gene expression data to minimize the chance of overfitting of the method and to improve the test accuracy. Thus, BPFS selects a biologically meaningful feature set that is minimally redundant. Our experiments on published breast cancer datasets demonstrate that all of the top 20 genes found by our method are associated with cancer. Furthermore, the classification accuracy of our signature is up to 18% better than that of vant Veers 70 gene signature, and it is up to 8% better accuracy than the best published feature selection method, I-RELIEF.

## 1. Introduction

 An important challenge in cancer treatment is to classify a patient to an appropriate cancer class. This is because class specific treatment reduces toxicity and increases the efficacy of the therapy [1]. Traditional classification techniques are based on different kinds of clinical markers such as the morphological appearance of tumors, age of the patients, and the number of lymph nodes [2]. These techniques however have extremely low (9%) prediction accuracies [3].

Class prediction based on gene expression monitoring is a relatively recent technology with a promise of significantly better accuracy compared to the classical methods [1]. These algorithms often use microarray data [4] as input. Microarrays measure gene expression and are widely used due to their ability to capture the expression of thousands of genes in parallel. A typical microarray database contains gene expression profiles of a few hundred patients. For each patient (also called observation), the microarray records expressions of more than 20 000 genes. We define an entry of a microarray as a *feature*.

Classification methods often build a classification function from a training data. The class labels of all the samples in the training data are known in advance. Given new sample, the classification function assigns one of the possible classes to that sample. However, as the number of features is large and the number of observations is small, standard classification algorithms do not work well on microarray data. One potential solution to this problem is to select a small set of relevant features from all microarray features and use only them to classify the data.

The research on microarray feature selection can be divided into three main categories: filter, wrapper, and embedded [5]. We elaborate on these methods in Section 2. These methods often employ statistical scoring techniques to select a subset of features. Selection of a feature from a large number of potential candidates is however difficult as many candidate features have similar expressions. This potentially leads to inclusion of biologically redundant features. Furthermore, selection of redundant features may cause exclusion of biologically necessary features. Thus, the resultant set of features may have poor classification accuracy.

One way to select relevant features from microarray data is to exploit the interactions between these features, which is the problem considered in this paper. More specifically we consider the following problem.

**Problem Statement**

Let *D* be the training microarray dataset where each sample belongs to one of the *T* possible classes. Let *P* be the gene regulatory and the signaling network. Choose *K* features using *D* and *P* so that these features maximize the classification accuracy for an unobserved microarray sample that has the same distribution of values as those in *D*.

**Contributions**

**Figure 1:**
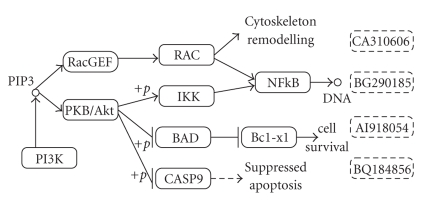
Part of Pancreatic cancer pathway adapted from KEGG showing the gene-gene interactions. → implies activation and ⊣ implies inhibition. The rectangles with solid line represent valid genes mapped to the pathway. They are referred to by the name of the genes. +*p* denotes phosphorylation. For example, PKB/Akt activates IKK through phosphorylation. IKK in turn activates NFkB. Thus, PKB/Akt indirectly activates NFkB. The rectangles with dotted lines are genetic sequence that do not have Entrez Gene ID and not mappable to pathway. We cannot yet associate them to some pathway. We denote them as *unresolved genes*. They are referred to by GenBank Accession numbers.

Unlike most of the traditional feature selection methods, we integrate gene regulatory and signaling pathways with microarray data to select biologically relevant features. On the pathway, one gene can interact with another in various ways, such as by activating or inhibiting it. In Figure 1, RacGEF activates RAC, BAD inhibits Bcl-xl, and PKB/Akt inhibits BAD by phosphorylation. We use the term *influence* to imply this interaction between two genes. We quantify influence by considering the number of intermediate genes between two genes on the pathway that connects them. The influence is the highest when two genes are directly connected. *Our hypothesis in this paper is that selecting two genes that highly influence each other often implies inclusion of biologically redundant genes.* The rationale behind this is that manipulating one of these genes will have significant impact on the other one. Thus, selecting one of them produces comparable prediction accuracy. So we choose the set of features such that each of them has the lowest influence on other selected features.

We propose a novel algorithm called *Biological Pathway-based Feature Selection* algorithm (BPFS) based on the above hypothesis that has the following characteristics. 

(1) Let the complete set of features be *G* and the set of already selected features be *S*. BPFS ranks all the features in *G* − *S* with an SVM-based scoring method MIFS [6]. The score quantifies the capacity of a feature to improve the already attained classification accuracy. BPFS ranks features in decreasing order of their scores. 

(2) BPFS chooses a small subset *C* of highly ranked features from *G* − *S* and evaluates the influence of every feature in *C* on the features in *S*. Finally, it selects the feature in *C* that has the lowest influence on the features in *S* and moves it to *S* from *G* − *S*. BPFS repeats this step for a fixed number of iterations.

We observe that a significant fraction of the gene entries in the microarray do not have any corresponding gene in the pathway. We use the term *unresolved genes* to represent these genes. We propose a probabilistic model to estimate the influence of those genes on selected features.

We tested the performance of our method on five breast cancer data sets [7–11] to predict whether breast cancer for those patients relapsed before five years or not. Our experiments show that our method achieves up to 18% and 8% better accuracy than the 70-gene prognostic signature [7] and I-RELIEF [2], respectively.

The organization of the rest of this paper is as follows. Section 2 discusses the background material. Section 3 describes the proposed algorithm. Section 4 presents experimental results. Section 5, briefly, concludes the paper.

## 2. Background

 Feature selection is an important area in data mining for preprocessing of data. Feature selection techniques select a subset of features to reduce relatively redundant, noisy, and irrelevant part of the data. The reduced set of features increases the speed of the data mining algorithms and improves accuracy and understandability of result. Feature selection is often used in areas such as sequence, microarray, and mass-spectra analysis [5]. The popular feature selection methods can be broadly categorized into the following.

**Filter Methods (see [12–14])**

These are widely studied methods that work independent of the classifier. They rank the features depending on the intrinsic properties of data. One such method is to select sets of features whose pairwise correlations are as low as possible.

**Wrapper Methods (see [15, 16])**

These methods embed the feature selection criteria into the searching of subset of features. They use a classification algorithm to select the feature set and evaluate its quality using the classifier.

**Embedded Methods (see [17])**

These approaches select features as a part of the classification algorithm. Similar to the wrapper methods, they interact with the classifier, but at a lower cost of computation.

All the above-mentioned traditional feature selection methods ignore the interactions of the genes. Considering each gene as an independent entity can lead to redundancy and low classification accuracy as many genes can have similar expression patterns.

Several recent works on microarray feature selection have leveraged metabolic and gene interaction pathways in their methods. Vert and Kanehisa [18] encoded the graph and the set of expression profiles into kernel functions and performed a generalized form of canonical correlation analysis in the corresponding reproducible Hilbert spaces. Rapaport et al. [19] proposed an approach based on spectral decomposition of gene expression profiles with respect to the eigenfunctions of the graph. Wei and Pan [20] proposed a spatially correlated mixture model, where they extracted gene specific prior probabilities from gene network. Wei and Li [21] developed a Markov random field-based method for identifying genes and subnetworks that are related to a disease. A drawback of the last two model-based approaches is that the number of parameters to be estimated is proportional to the number of genes. So optimizing the objective function is costly as the number of genes in microarrays is more than 20 000. C. Li and H. Li [22] introduced a network constraint regularization procedure for linear regression analysis, which formulates the Laplacian of the graph extracted from genetic pathway as a regularization constraint.

 One limitation of all the above mentioned methods that use biological pathway is that, all of them consider genetic interactions between immediate neighbors on the pathway. None of them explicitly consider interactions that are beyond immediate neighbors. Also, most of them performed quantitative analysis of the selected features on simulated datasets. So it is not possible to quantify the accuracy of those selected features on some real datasets only from the results in these papers. Additional experiments on real microarray datasets are required to justify those methods and their set of features. Also, as the reconstruction of genetic pathway is yet to be completed, we cannot always map a microarray entry to the biological pathway. They do not consider the implications of those missing information. In this paper, we introduce a new microarray feature selection method that addresses these issues.

## 3. Algorithm

This section describes our Biological Pathway-based Feature Selection algorithm (BPFS) in detail. BPFS takes a labeled two-class microarray data as input and selects a set of features. Algorithm 1 portrays a synopsis of BPFS. We discuss an overview of BPFS next.

We denote the set of all features by *G*. Let *S* be the set of features selected so far. The set *G* − *S* represents all the remaining features. BPFS iteratively moves one feature in *G* − *S* to *S* using the following steps, till the required number of features is selected (along with their rank).

**Step 1 . (determine the *t* best candidates (see 2(a) to 2(b) in Algorithm 1))**

This step creates a candidate set of features from *G* − *S* by considering their classification accuracy alone. To do this, BPFS first sorts all the available features in decreasing order of their *marginal classification power* and chooses the top *t* (typically *t* = 10 in practice) of them as the candidate set for next step. We define the marginal classification power of a feature as its ability to improve the classification accuracy when we include it into *S*. Let us denote the set that contains these top *t* features by the variable *C*.

**Step 2 . (pick the best gene using pathways (see 2(c) to 2(d) in Algorithm 1))**

In this step, we use signaling and regulatory pathways to distinguish among the feature set *C* obtained in Step 1. Given a set of already selected features *S*, BPFS aims to select the next most biologically relevant feature from *C*. We define a metric to compare the features in *C* for this purpose. This metric estimates the total *influence* between a candidate feature and the set of selected features. We denote this total influence as the *Total Influence Factor *(TIF). TIF is a measure of the potential interaction (activation, inhibition, etc.) between a candidate gene and all the selected genes. A high value of TIF for a gene implies that the gene is highly influenced by some or all of the already selected set of genes. We choose the gene in *C* that has the lowest TIF. We then include it in *S*. We elaborate this step in Section 3.3.

In the following subsections we discuss the above aspects of our algorithm in more detail. Section 3.1 defines how we select our first feature. Section 3.2 discusses the first round of selection procedures based on classification capability. Section 3.3 describes the use of pathways for feature selection. Section 3.4 presents a technique to utilize the training space efficiently in order to improve the quality of features.

### 3.1. Picking the First Feature: Where to Start?

BPFS incrementally selects one feature at a time based on the features that are already selected. The obvious question, then is, how do we select the first feature? There are many alternative ways to do this. One possibility is to get an initial feature using domain knowledge. This, however, is not feasible if no domain knowledge exists on the dataset.

We use mutual information to quantify the discriminating power of a feature. Let us represent the *k*th feature of microarray using a random variable *F* and the class label of the data using another random variable *L*. Assume that there are *n* observations in the data. *F* and *L* can assume different values over those *n* observations. Let *f* an instance of *F* and *l* be an instance of *L*. The mutual information of *F* and *L* is *I*(*F*, *L*) = ∑_*f*∈*F*,*l*∈*L*_ *ψ*_*F*,*L*_(*f*, *l*)log (*ψ*_*F*,*L*_  (*f*, *l*)/*ψ*_*F*_  (*f*)*ψ*_*L*_  (*l*)), where *ψ*_*F*,*L*_ is the joint probability mass function of *F* and *L*; *ψ*_*F*_ and *ψ*_*L*_ are the respective marginal probability mass function of *F* and *L*. Thus, we use *I*(*F*, *L*) to quantify the relevance of the *k*th feature for classification. We choose the feature with maximum mutual information as the starting feature.

Another way to select the first feature can be to utilize the marginal classification power. Essentially, it is way we can apply the second step of our algorithm which *S* = {} and select the top candidate as the first candidate.

Next we discuss how we select the remaining features.

### 3.2. Selecting the Candidate Features

In this step, BPFS sorts all the available features in *G* − *S* in decreasing order of their *marginal classification power*. We define the marginal classification power of a feature later in this section. BPFS then chooses the *t* features with the highest marginal classification power as the candidate set that will be explored more carefully in the subsequent steps. We elaborate on this next.

We use an SVM based algorithm, MIFS [6], to calculate the *marginal classification power* of all available features as follows. BPFS, first, trains SVM using the features in *S* to get the value of the objective function of SVM. We use linear kernel for the SVM in our experiments. For very high dimensional data a linear kernel performs better than or comparable to a non-linear kernel [23]. A linear kernel is a simple dot product of the two inputs. So the objective function of SVM becomes

(1) $$\begin{array}{c} J=\overset{n}{\underset{i=1}{\sum }}\alpha_{i}-\frac{1}{2}\overset{n}{\underset{i,j=1}{\sum }}\alpha_{i}\alpha_{j}y_{i}y_{j}x_{i}\cdot x_{j} \end{array},$$

where *α*_*i*_, *y*_*i*_ and *x*_*i*_ denote the Lagrange multiplier, the class label and the value of selected set of features of *i*th observation respectively. Here, *x*_*i*_ *x* − *j* are vectors and *x*_*i*_ · *x*_*j*_ is the dot product of them. Then for each feature *m*∈*G* − *S*, BPFS calculates the objective function *J* if *m* is added to *S* as

(2) $$\begin{array}{c} J\left(S\cup \left\{m\right\}\right)=\overset{n}{\underset{i=1}{\sum }}\alpha_{i}-\frac{1}{2}\overset{n}{\underset{i=1,j=1}{\sum }}\alpha_{i}\alpha_{j}y_{i}y_{j}x_{i}\left(+m\right)\cdot x_{j}\left(+m\right) \end{array}.$$

Here *x*_*i*_( + *m*) denotes the value of the selected set of features along with the aforementioned feature *m* for the *i*th observation. Using the last two equations, we calculate the marginal classification power of a feature *m*, as the change in the objective function of SVM, when *m* is included in *S*. We denote this value with variable Δ*J*. To paraphrase, marginal classification power of a feature is the capability of a new feature to improve the classification accuracy of a set of selected features, when the new feature is added to the already selected set. Formally, we compute Δ*J*(*m*) for all *m*∈*G* − *S* as Δ*J*(*m*) = *J*(*S*∪{*m*}) − *J*(*S*). BPFS sorts all the features *m* ∈ *G* − *S* in descending order of Δ*J*(*m*). It considers the top *t* (*t* = 10 in our experiments) genes as possible candidates for the next round. Let *C* denote the set of these *t* genes. In the next steps, BPFS examines biological networks to find out the most biologically meaningful feature in *C*.

### 3.3. Selecting the Best Candidate Gene

All the features in the candidate set *C* often have high marginal classification power. In this step, we distinguish the features in *C* by considering their interactions with the features in *S* (the set of features that are already selected). We hypothesize that if a feature in *C* is influenced by the features in *S* greatly, then that feature is redundant for *S* even if it has high marginal classification power. We discuss how we measure the influence of a feature on another one next.

Consider the entire pathway as a graph, where all the genes are vertices and there is an edge between two vertices if they interact with each other. In this paper, we do not consider any specific pathway such as p53 signaling pathway, rather a consolidation of all the available human signalling and regulatory pathways. If we have had the knowledge about the pathways that are affected by that specific biological condition (such as cancer), we could select features only from those pathways. However, the available literature does not provide the comprehensive list of affected pathways most of the time. Thus, we create a consolidation of all the regulatory and signaling pathways.

There are different kinds of interactions such as activation and inhibition. If two genes do not have a common edge, but they are still connected by a path, it means that they interact indirectly through a chain of genes. For example, in Figure 1, RacGEF activates RAC and RAC activates NFkB. Thus, RacGEF *i* *n* *d* *i* *r* *e* *c* *t* *l* *y* activates NFkB through RAC. We, therefore, compute the distance between them as two. A higher number of edges on the path that connects two genes implies feebler influence.

An abnormally expressed gene does not necessarily imply that its neighbor will be abnormally expressed [24]. This is because the interaction between two genes is a probabilistic event. For example, in Figure 1, if RacGEF becomes aberrantly expressed, there is a probability that RAC is also expressed aberrantly. Let us denote this probability with the variable *h*. Similarly, if RAC is abnormally expressed, NFkB is abnormally expressed with a probability *h*. So, if RacGEF is over expressed, NFkB can be over expressed with a probability of *h* ^2^. This leads to the conclusion that as the number of hops increases the influence decreases exponentially. Thus, we use an exponential function to model this influence.

To quantify influence we define a metric, termed *Influence Factor *(IF), between two genes *g*_*i*_ and *g*_*j*_ as IF(*g*_*i*_, *g*_*j*_) = 1/2^*d*(*g*_*i*_,*g*_*j*_)−1^, provided *i* ≠ *j*. Here *d*(*g*_*i*_, *g*_*j*_) is the length of the shortest path that connects *g*_*i*_ and *g*_*j*_ on the pathway. To calculate total influence on a candidate gene asserted by a selected set of genes we calculate IF between the candidate gene and every gene in the selected set and sum it up. We call it the *total influence factor *(TIF) of a candidate gene *g* with respect to already set of selected genes. Formally,

(3) $$\text{TIF}\left(g,S\right)=\underset{s\in S}{\sum }\frac{1}{2^{d(g,s)-1}}$$

*d*(*g*, *s*) is zero if there is no path between *g* and *s*.

For example, in Figure 1 consider PKB/Akt as a candidate gene and assume that *S* consists of two genes NFkB and CASP9. CASP9 is one hop away from PKB/Akt. So IF(PKB/Akt, CASP9) = 1. The shortest path from PKB/Akt to NFkB is of two hops through IKK. So IF(PKB/Akt, NFkB) = 0.5. Thus, TIF(PKB/Akt, {CASP9, NFkB}) = 1.5.

BPFS calculates TIF for all the genes in *C* and selects the one that generates the lowest value of TIF. A low TIF value implies lower aggregate influence on the set of selected features *S*. To paraphrase, the gene with lowest TIF is least interacting with the genes in *S*. So, we select the gene that is biologically most independent from *S*.

The gene databases, like KEGG, are still evolving. Thus, many of the genes cannot be mapped from microarray data to these databases. In Section 4 we describe a probabilistic technique to handle this problem.

### 3.4. Exploration of Training Space

We have described the key components of our feature selection algorithm (BPFS) in the previous subsections. As a dataset consists of comparatively smaller number of observations and a large feature set, BPFS is prone to overfitting. To avoid this problem, we propose a method that utilizes the training space efficiently.

Let *D*_*T*_ be the training data. We create *K* data subsets (*K* = 50 in our experiments) *D*_*T*_1__,*D*_*T*_2__,…,*D*_*T*_*K*__ each containing 80% of the *D*_*T*_ randomly sampled from it. We, then, run BPFS on each of them and get *K* sets of features. We store these *K* feature sets in a *K* × *N* matrix *M*, where the *i*th row contains first *N* features obtained from *D*_*T*_*i*__. Thus, *m*_*ij*_ is the *j*th feature obtained from *D*_*T*_*i*__. We use this matrix to rank all the features in the following fashion.

1. We assign a linearly decreasing weight across a row to emphasize the importance of the features that come first. More specifically, we assign a weight of *N* − *k* to a feature that appears in the *k*th column of a row.
2. We sum the weights of the features over all the rows to determine the overall weight of the features in *M*. For example, assume that a feature appears in three rows of *M*, at (5,3), (17,14), and (29,10), where the first number in each pair indicates the row and second number indicates the column. Also, assume that we want to choose a total of 150 features. Then, the total weight of this feature is (150 − 3) + (150 − 14) + (150 − 10) = 423.

We pick the *N* features with the highest weight from our feature set. Weighing the features based on their positions helps us to prioritize the features that occur frequently and/or appear with high rank. We discuss the impact of the value of *N* in Section 4.

## 4. Data Set and Experiments

 In this section we evaluate BPFS experimentally. We use multiple real microarray datasets instead of synthetically generated data, as synthetic data may not accurately model different aspects of a real microarray data [25]. We observe that we can map only a small portion (25%) of the microarray entries to KEGG regulatory pathway. Some of them do not take part in any single interaction. So the only information we have about them is their measured expression value on the microarray dataset. Due to this missing data problem it is difficult to quantify the implication of biological pathway in our algorithm. To handle this problem we have conducted our experiments on two different kind of information. In one case, we use the KEGG pathways as it is and used a randomized technique to handle the interactions with unresolved genes. In the other case, we map all the microarray genes to KEGG pathway and assume that genes within a single pathway are fully connected and there is no common gene between two pathways. Still, we need to be careful while interpreting the results with fully connected pathway as it is only a simplistic view of the actual pathway. We cover the experiments with real pathways in the paper from Sections 4.2–4.6. In Section 4.7 we discuss the experiments with fully connected pathways.

In Section 4.1 we describe the experimental setup. In Section 4.2 we describe the randomization technique. We show the biological validity of our feature set, by tabulating the supporting publications against every feature (Section 4.3). We compare our signature against van't Veer's [7] on four data sets (Section 4.4). We compare the testing accuracy of our method to that of I-RELIEF, a leading microarray feature selection method (Section 4.5). We conducted cross-validation experiments where we extracted features from one dataset and tested its accuracy on another dataset in Section 4.6. Finally, we executed BPFS and I-RELIEF on an idealistic fully connected pathway in Section 4.7.

### 4.1. Experimental Setup

**Microarray Data**

In our experiments we used five breast cancer microarray datasets from the literature. We name these datasets as BCR [10], JNCI [8], Lancet [9], CCR [12] and Nature [7], respectively, according to the name of the journals they were published. BCR, CCR, and Lancet use Affymetrix GeneChip Human Genome U133 Array Set HG-U133A consisting of 24, 481 entries. Nature has its own microarray platform with 24, 481 entries. JNCI has the same platform as that of Nature, but it consists of a much smaller feature set of 1, 145 entries. We removed the observations whose class labels were not defined. For the rest of the data points we created two classes depending on whether relapse of the disease happened in five years or not, counting from the time of the primary disease. The datasets Nature, JNCI, BCR, CCR, and Lancet contain 97, 291, 159, 190, and 276 observations, respectively.

**Pathway Data**

We used the gene regulatory and signaling pathways of * Homo Sapience* in KEGG. We merged all the relevant pathway files to build a consolidated view of the entire pathway. The final pathway consists of 8 270 genes and 7 628 interactions. Clearly, some genes do not take part in any interaction.

**Training and Testing Data**

We randomly divided a microarray dataset (e.g., BCR dataset) in 4  :  1 ratio to create training and testing subset. We maintained the distribution of two classes in the undivided dataset unchanged in the training and testing subset. We collected features from the training dataset and tested the classification accuracy using those features on the test dataset. Now we elaborate on how we utilized the training space to select features. We created a number of subsets *K* (typically 50 in our experiments) using bootstrapping from training dataset. Each subset contains 80% samples of the training data. We selected features from each of those subspaces using Section 3.1 to Section 3.3. Then we combined the *K* obtained set of features using the method of Section 3.4.

**Implementation and System Details**

We implemented our feature selection algorithm (BPFS) using Matlab. For SVM, we used “Matlab SVM Toolbox”, a fast SVM implementation in C based on sequential minimal optimization algorithm [26]. For pathway analysis code we used Java. We ran our implementation on a cluster of ten Intel Xeon 2.8 GHz nodes on Ubuntu Linux.

**Availability of code**

The implementation of the proposed method can be downloaded from http://bioinformatics.cise.ufl.edu/microp.html.

### 4.2. Pathways with Unresolved Genes

To calculate the influence factor (IF) we need to calculate the number of hops between two genes on the pathway. This requires a mapping of those microarray entries to pathway genes. However, as some of the microarray entries are not complete genes and biological pathway construction is not yet finished [59], we can not map all microarray entries. We denote all the unmapped genes as *unresolved genes*. For example, Affymetrix microarray HG-U133A contains 24,481 entries. We are able to map only 6 500 entries to KEGG (Kyoto Encyclopedia of Genes and Genomes). For example, in Figure 1 we draw four rectangles with dotted lines that correspond to four microarray entries in Affymetrix platform. As they do not have Entrez Gene identification number, we can not associate them with any pathway. Hence, these are unresolved genes.

Our preliminary experiments suggest that unresolved genes represent a large fraction of the genes in set *C* (more than 60% on average). To estimate the TIF of the unresolved genes, we develop a probabilistic model. Let *C* be the set of candidate genes and *S* be the set of selected genes in an iteration of BPFS. While calculating TIF for a *g* ∈ *C* we consider two cases:

**Case 1 : (the candidate gene is resolved)**

Assume that *g* is resolved. Let *Q*⊆*S* be the set that contains all the unresolved features in *S*. Let *R* = *S* − *Q* be the set of resolved features in *S*. Let *p* be the expected influence of a gene *q* ∈ *Q* on gene *g* if all genes were mapped and the pathway construction was complete. We discuss how we estimate the value of *p* later in this section. Then the expected number of genes from *Q* having influence on *g* is TIF(*g*, *Q*) = *p* · | *Q*|, where |*Q*| is the number of genes in *Q*. So the Total Influence Factor becomes

(4) $$\begin{array}{cc} \text{TIF}\left(g,S\right) & =\text{TIF}\left(g,Q\right)+\text{TIF}\left(g,R\right)\\ & =p\left|Q\right|+\underset{s\in R}{\sum }2^{1-d(g,s)}. \end{array}$$

**Case 2 : (the candidate gene is unresolved)**

When *g* is unresolved we consider it as a special case of Case 1. As the connectivity between *g* and all genes in *S* is unknown, we estimate TIF as

(5) $$\begin{array}{c} \text{TIF}\left(g,S\right)=p\left|S\right| \end{array}.$$

In summary, to handle the unresolved genes we augment the probabilistic model to biological pathway-based selection and replaced (3) by (4). Among the genes in candidate set *C* we select the gene with smallest TIF using (4). Now, we describe how we derive the value of *p* in (4) and (5).

To derive the value of *p*, we propose the following approach. It is reasonable to assume that there are many missing interactions in the currently available pathway databases, since missing interactions are continually being discovered. Let us denote the present incomplete pathway graph by *P*_*I*_ and the hypothetical complete pathway by *P*_*C*_. Assume that *P*_*C*_ contains *z* times more interactions than that of *P*_*I*_. From *P*_*I*_ we estimate *p* as a function of *z* and the expected number of the genes in *P*_*I*_ as follows. We, first, build a graph *P*_*I*_ as described in Section 3.3 from the KEGG database. We, then, randomly delete edges of *P*_*I*_ and create the subgraphs *P*_10_, *P*_20_,…, *P*_100_ of *P*_*I*_ corresponding to 10%, 20%,…, 100% of edges of that of *P*_*I*_. For each of these sub-graphs we calculate the average number of vertices reachable from a vertex.

Formally, let *V* denote the set of vertices in *P*_*s*_, where *s* ∈ {10,20,…, 100}. We denote the number of reachable vertices from *g* (*g* ∈ *V*) by *R*(*g*). Then, the average reachability of *P*_*s*_ is

(6) $$\begin{array}{c} R_{P_{s}}=\frac{\sum_{g\in V}R\left(g\right)}{\left|V\right|} \end{array},$$

where |*V*| is number of vertices in *V*. We calculate *R*_*P*_10__,…, *R*_*P*_100__, the average reachability for all the subgraphs.

**Figure 2:**
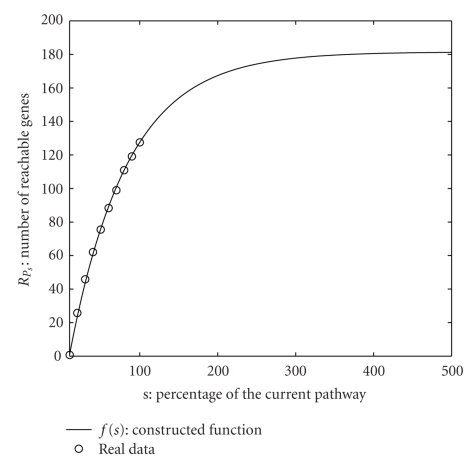
We plot the real data points *R*_*P*_10__,…, *R*_*P*_100__ along with the constructed function *f*(*s*) against s, the fraction of the current pathway. We extrapolate *f*(*s*) up to *s* = 500. *f*(*s*) converges around 180.

We, then, construct the function *f*(*s*) that evaluates to *f*(*s*) = *R*_*P*_*s*__. To construct *f*(*s*), we use a converging power series. We derive the value of parameters using *R*_*P*_10__,…, *R*_*P*_100__. To calculate the average reachability of the hypothetical pathway *P*_*C*_ we use value of s greater than 100 in *f*(*s*). In Figure 2 we plot *R*_*P*_10__,…, *R*_*P*_100__ along with the constructed function *f*(*s*) against the s, the fraction of the current pathway.

We observe that *f*(*s*) interpolates the values *R*_*P*_10__,…, *R*_*P*_100__ accurately and it converges at around *s* = 500 with the value of 180. Thus, we can conclude that the average reachability of *P*_*C*_ is around 180. The probability that an unresolved gene has an interaction with a randomly chosen gene in *S* in *P*_*C*_ is given by *p* = *R*_*P*__*c*_/| *V*| where |*V*| is number of nodes in the pathway. As the total number of human genes is close to 20,500 [60], we get 0.0088 as the value of *p*.

### 4.3. Biological Validation of Selected Features

We collected the list of publications that support the relevance of the first twenty features selected from BCR on cancer. Table 1 lists the publications and cancer types for each of the genes.

To get the features, we created the training dataset as described in Section 4.1. We, then, trained BPFS on the training data and obtained a ranked set of features. We repeated this process ten times on BCR. We selected first twenty features from each rankings and merged them using the method described in Section 3.4 to get the final twenty features. We found relevant publication for all the twenty genes. We observed that four of them are directly responsible for breast cancer. Another seven genes are associated to breast cancer from the point of histology (two prostrate, four gastric and one colorectal). The rest of them are related to other kinds of cancer such as ovarian cancer and lung cancer.

In some cases a gene is involved for more than one kind of cancer. For example, ASCL1 is associated with both prostrate cancer and lung cancer. We concluded that BPFS chooses the set of genes that are responsible for breast cancer and other kind of cancers. *Hence, BPFS selects a biologically meaningful feature set that reduces the number of redundant features and improves generalization accuracy by selecting more relevant feature set.*

In general, the above approach of combining features obtained from ten different runs may lead to selecting features from the entire dataset. We did it only for this experiment to filter out the biologically significant features from a dataset. For the remaining experiments we kept separate training and testing datasets.

### 4.4. Comparison with van't Veer's Gene Signature

In this section we compare our gene signatures to the breast cancer prognostic signature found by van't Veer et al. [7]. van't Veer et al. generated the 70 gene signature using a correlation based classifier on 98 primary breast cancer patients. van't Veer's 70 gene experiment [7] demonstrated that genetic signature can have a much higher accuracy in predicting disease relapse free survival against clinical markers (50% versus 10%).

**Figure 3:**
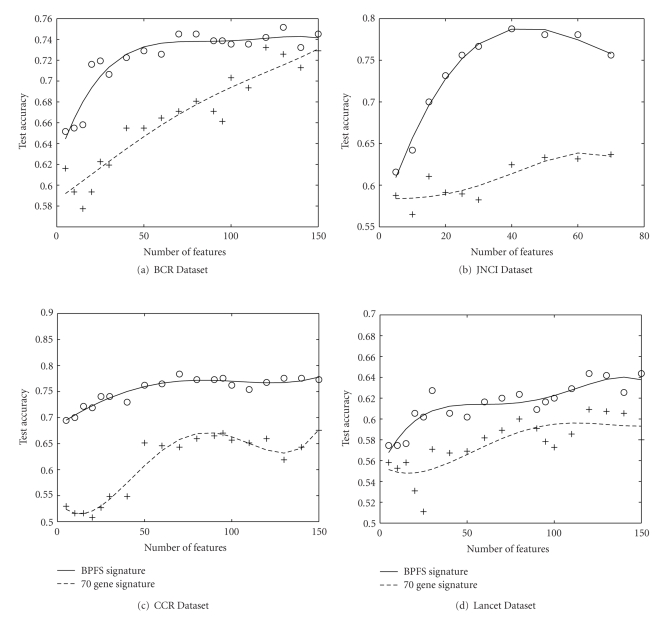
Comparison of test accuracy of our signature and van't Veer's 70-gene prognostic signature on real pathway. For all the datasets, our signature performs significantly better than their signature.

In our experiment, we demonstrate that our method finds a better gene signature than these 70-gene signature for a particular dataset. We created training and testing data from the four datasets JNCI, Lancet, BCR and CCR as described in Section 4.1. From those four training dataset we created four set of features using our method. We calculated the accuracy of the four set of features using the corresponding four test datasets. Also, we computed the test accuracy using this 70-gene signature on the same four test dataset just mentioned. Finally we plotted the testing accuracies obtained from both set of features in Figure 3.

Figure 3 illustrates the results for up to 150 genes for both the signature on BCR, CCR and Lancet. We observe that for all four data sets our accuracy is better than that of 70-gene signature. BPFS attains 18% better accuracy for JNCI dataset. From this we conclude that BPFS finds a better gene signature for all the datasets.

### 4.5. Comparison with I-RELIEF

We compare the accuracy of our method to that of I-RELIEF [2]. I-RELIEF is a nonlinear feature selection algorithm. It produced significant accuracy over van't Veer's 70 gene prognostic signature and standard clinical markers [61].

**Figure 4:**
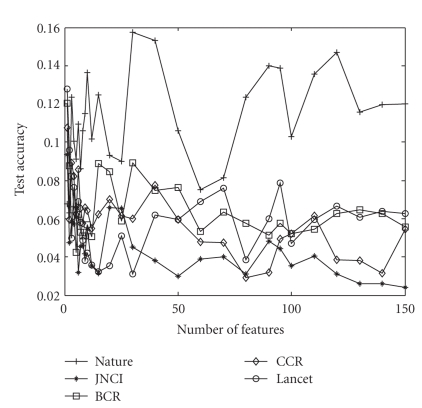
The standard deviation of the accuracy of our method for different number of features. The *x*-axis denotes different number of features.

We, first, created training and testing dataset from the given data as discussed in Section 4.1. We obtained the ordered feature set by training the BPFS on the training data. We tested the quality of those features using the SVM classifier. We used identical set up and data sets for I-RELIEF. We used 2.0 as the kernel width for I-RELIEF as recommended [2]. We repeated the experiments ten times on each data set and present the average accuracy for different features. Figure 4 plots the standard deviation of the accuracy of our method over this 10 times of running. For all of them except the Nature dataset, the standard deviation is less than 1 (i.e., 10% accuracy). So we can conclude that our method is quite stable while we execute it over several subsets of data created from a single dataset.

**Figure 5:**
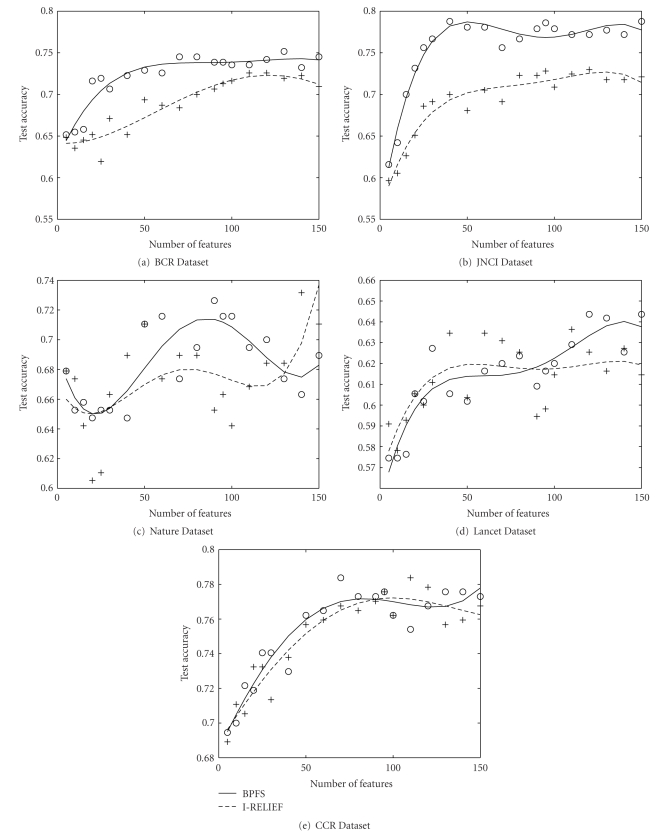
Comparison of test accuracy of our method (BPFS) to that of I-RELIEF on real pathway. In three datasets JNCI, BCR and Nature our method performs better than I-RELIEF. In Lancet and BCR both the methods have similar accuracy.

Figure 5 compares our algorithm to I-RELIEF. We observe that for two data sets (BCR and JNCI), BPFS outperforms I-RELIEF for all the features selected. BPFS shows highest improvement (8%) over I-RELIEF for JNCI dataset, at around 50 features. JNCI has higher fraction of resolved genes (45% versus 25%). Thus, BPFS has a higher chance to select more resolved genes. This implies less dependability on the probabilistic model, the selection of genes is more accurate. From this observation, we expect that BPFS would produce better result when the missing links of the pathway would be discovered. For Nature dataset, BPFS produces better accuracy than I-RELIEF up to 130 features. BPFS has similar accuracy with that of I-RELIEF for CCR and Lancet data.

Our algorithm is based on linear kernel which is in general more appropriate when the number of features is much higher than number of samples. On the other hand I-RELIEF employs a non-linear kernel [23]. It is possible that the distribution of Lancet data works better with the type of kernel that I-RELIEF uses. We can potentially improve the classification accuracy of BPFS by using a non-linear kernel. 

We observe that for all the datasets our method reaches its highest accuracy at around 50–70 features. We conclude that these 50–70 features consist the most important set of genes that are associated with the breast cancer.

### 4.6. Cross Validation Experiments

We conduct several cross validation experiments where we generate a set of features on one data set and validate its quality by testing it on some other data set. For this cross validation, we limit ourselves to the same microarray platform that we use to generate the feature set. For example, we test BCR dataset's features on Lancet as the microarray platforms on which they were generated are same. The main reason for doing so is that the set of genes used in two different microarrays can be different. Even for the same gene they use different part of the genomic sequence. Thus, inter-platform validation may not be representative of the actual generalization. Table 2 displays the result of our cross validation experiments.

We use two different version of our algorithm, one with the pathway information and another without the pathway information on observe the contribution of inclusion of the pathway information into our algorithm. In Table 2 we denote the version of our method with pathway by putting 1 at superscript of the result. Similarly we denote the version without pathway by putting 2 at the superscript of the datasets.

Also, to establish the relevance of our signature on a standard benchmark, we compare our signatures with van't Veer's 70-gene signature in the context of cross validation. For example, in Table 2 BCR dataset is cross validated with three feature sets obtained from Lancet, CCR and 70-gene signature. We observe that on an average our signatures perform better than the 70-gene one. For CCR data, both Lancet and BCR features generate better accuracy. For Lancet data the accuracies obtained using CCR and BCR features are similar to that of 70-gene signature. For BCR data, up to 80 features of our signatures outperform the 70-gene signature. Beyond that the 70-gene signature has a better accuracy. To sum up, we get better accuracy while testing with the features on a different platform compared to van't Veer's prognostic signature.

 Regarding the comparison of the two versions of our algorithm (with and without the pathway information) it's difficult to reach a conclusive decision. For example, while we extract the feature set from CCR dataset and cross validate it BCR dataset the algorithm without pathway information is doing better than the other for upto 20 features, but for the larger number of features the algorithm with pathway information provides a better accuracy. 

When we compare the accuracy with features from a different dataset to that of its own feature set, we observe that on the average, the drop of the accuracies are not more than 6%. For some extreme cases the drop can be higher. Specifically when we cross validate with features extracted from Lancet, the accuracy is lower.

### 4.7. Experiments on Fully Connected Pathways

**Figure 6:**
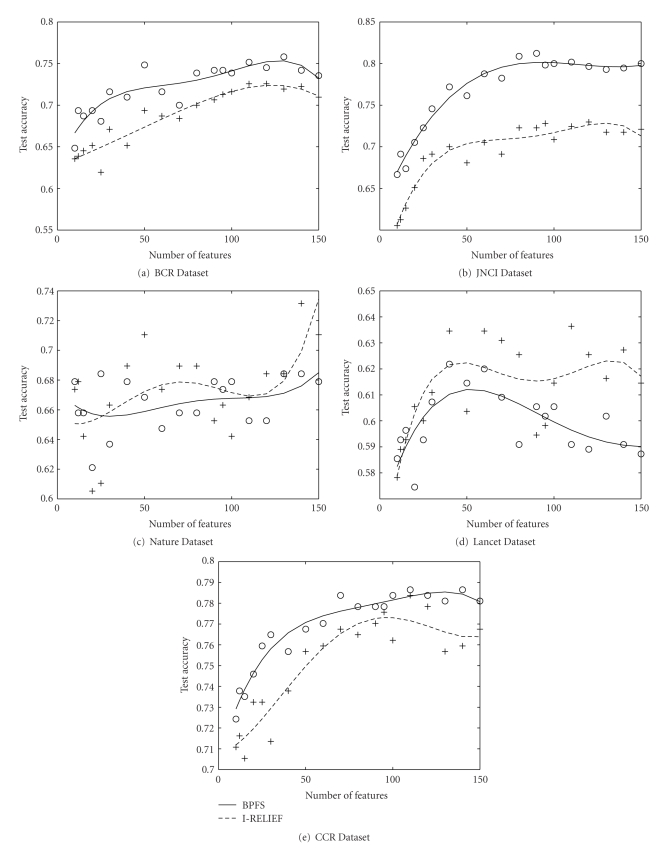
Comparison of test accuracy of our method (BPFS) to that of I-RELIEF on fully connected pathway. In three datasets JNCI, BCR and CRR our method performs better than I-RELIEF. In Lancet and BCR I-Relief has a better accuracy.

In this section we describe the experiments with idealistic fully connected pathway. Here, the approach we took was to evaluate our experiments on an idealistic pathway, where we map all the genes including the unresolved genes into the KEGG pathway. The unmapped genes become singleton pathway with only a single member gene. For other pathways that are already listed in the KEGG database we assume that all the genes within a pathway are fully connected and there is no connection between two pathway. If we consider each pathway as the smallest indivisible module of interactions, then all the genes within a pathway coexpress in a similar fashion. We compare the accuracy of BPFS with this pathway with that of I-RELIEF. In Figure 6 we see that for BCR, JNCI and CCR environment, BPFS has a better accuracy. For BCR there is an improvement of 5% around when BPFS uses around 40 features. For JNCI, the improvement is over 10% with around 70 features. For CCR there is a 3% improvement for 30 features. For Nature dataset, I-RELIEF has slightly (around 1.5%) better accuracy up to 100 features. For Lancet dataset I-RELIEF performs better than BPFS. So, we observe that for three dataset BPFS has better accuracy, for one dataset the accuracy is almost comparable.

### 4.8. Contribution of Pathway Information

In this section, we describe the experiments that we conduct to understand the contribution of pathway information in our algorithm in terms of classification accuracy. In one version we use the complete version of the algorithm as it is, in the other version we select the features only based on the marginal classification power and skip the next step. 

**Figure 7:**
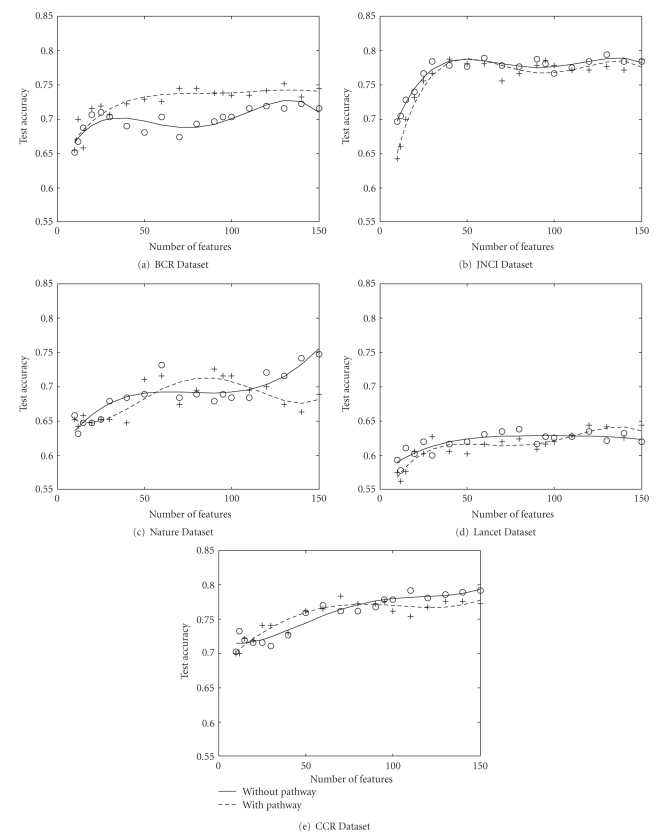
Comparison of test accuracy of our method (BPFS) to when we select the genes only based on marginal classification power.

 However, we observe that the contribution of biological network is not very decisive. For some dataset and some set of features the complete version of the method generates better accuracy, sometimes the trimmed version produces more accurate result. For instance, in Figure 7, for BCR dataset the complete method has upto 6% higher accuracy, where for Nature dataset the method with pathway generates better result for 50–100 number of features. The reason behind this fluctuation of accuracy might be that reconstruction of gene regulatory and signaling network is still in progress. Among almost 22 000 Affymetrix transcripts, we could map only 3300 genes that take part into at least one KEGG pathway. Even for those genes, the pathway construction is not complete. We hope that our algorithm can generate better accuracy in the near future when we can have a more comprehensive pathway structure.

## 5. Conclusions

 In this paper we considered feature selection problem for a classifier on cancer microarray data. Instead of using the expression level of a gene as the sole feature selection criteria we also considered its relation with other genes on the biological pathway. Our objectives were to develop an algorithm for finding a set of biologically relevant features and to reduce the number of redundant genes. The key contributions of the paper are the following.

- We proposed a new feature selection method that leverages biological pathway information along with classification capabilities to reduce the redundancy in gene selection based on biological pathway.
- We proposed a probabilistic solution to handle the problem of *unresolved genes* that are currently not mappable from microarray to biological pathway.
- We presented a new framework of utilizing the training subspace that improve the quality of feature set.

Our algorithm improve quality of features by a biological way by excluding the features that have total influence factor, and includes genes that are apart in the biological network and still have high marginal classification power. Thus, we believe that instead of selecting a close set of genes as features our method identify biologically important key features for a significant number of pathways. We demonstrated the biological significance of our feature set by tabulating the relevant publications. We also established the quality of our feature set by cross validating them on other data sets and comparing them against van't Veer's 70-gene prognostic signature. Our experiments showed that it is better than best published available method I-RELIEF.

## Figures and Tables

**Algorithm 1:**
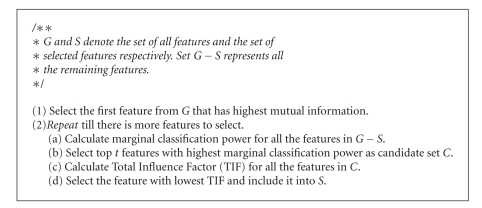
Biological Pathway-based Feature Selection Algorithm (BPFS).

**Table 1:** List of publications supporting the first twenty features obtained from BCR data set about their responsibility for cancer.

Gene	Supporting Publications	Gene	Supporting Publications
KCNK2	Acute Lymphoblastic Leukemia: [27]	ZNF222	Breast cancer: [28]

P2RY2	Human lung epithelial tumor: [29], Non-melanoma skin cancer: [30], Thyroid cancer: [31]	SLC2A6	Human Leukemia: [32]

CD163	Breast Cancer: [33], Human colorectal cancer [34]	HOXC13	Acute myeloid leukemia: [35, 36]

PCSK6	Breast Cancer: [37], Ovarian cancer: [38]	AQP9	Leukemia: [39]

PYY	Peptide YY (PYY) is a naturally occurring gut hormone with mostly inhibitory actions on multiple tissue targets [40]	KLRC4	KLRC4 is a member of the NKG2 group that are expressed primarily in natural killer (NK) cells [41]

CYP2A13	Lung adenocarcinoma: [42]	GRM2	Metastatic Colorectal Carcinoma: [43]

PHOX2B	Neuroblastoma: [44]	ASCL1	Prostate cancer: [45], Lung cancer: [46]

PKD1	Polycystin-1 induced apoptosis and cell cycle arrest in G0/G1 phase in cancer cells [47]. PKD1 inhibits cancer cells migration and invasion via Wnt signaling pathway in vitro [48]. Gastric cancer: [49]	ANGPT4	Gastrointestinal stromal tumor, leiomyoma and schwannoma [50], renal epithelial and clear cell carcinoma [51]

PSMB1	Breast Cancer: [52]	RUNX1	Gastric cancer: [53, 54], Ovarian cancer: [55], Classical tumor suppressor gene: [56]

CD1C	Prostate cancer: [57]	ZNF557	Myeloid Leukemia: [58]

**Table 2:** Accuracy of our algorithm obtained from Cross Validation experiments on real pathway. Feature set obtained from one data set is tested against another data set. We always chose target data from the same class of microarray in order to avert cross platform problems. The cross validation results implies that the feature set generated by BPFS provides satisfactory performance in cross data sets without significant loss of accuracy. We also compare our method with a trimmed version where we skip step 3.3. The complete version of the algorithm (with pathway) is indicated by 1 at the superscript while the trimmed version (without pathway) is denoted by the superscript 2.

Dataset used	Dataset used to	Number of Features								
for testing	extract the features	5	10	20	40	60	80	100	120	140
	BCR^1^	0.65	0.65	0.72	0.72	0.73	0.75	0.74	0.74	0.73
BCR	CCR^1^	0.63	0.61	0.61	0.63	0.66	0.68	0.66	0.71	0.69
	CCR^2^	0.64	0.62	0.65	0.63	0.64	0.65	0.66	0.65	0.68
	Lancet^1^	0.57	0.59	0.58	0.63	0.61	0.61	0.65	0.65	0.67
	Lancet^2^	0.55	0.55	0.63	0.62	0.58	0.64	0.66	0.69	0.7
	70-Gene-Sig^1^	0.62	0.59	0.59	0.66	0.67	0.68	0.70	0.73	0.71
	CCR^1^	0.70	0.70	0.72	0.73	0.77	0.77	0.76	0.77	0.78

CCR	BCR^1^	0.60	0.63	0.67	0.65	0.65	0.66	0.68	0.66	0.66
	BCR^2^	0.53	0.65	0.65	0.66	0.61	0.67	0.69	0.68	0.65
	Lancet^1^	0.57	0.58	0.62	0.60	0.66	0.69	0.68	0.70	0.71
	Lancet^2^	0.58	0.58	0.61	0.65	0.68	0.73	0.78	0.75	0.75
	70-Gene-Sig^1^	0.53	0.52	0.51	0.55	0.65	0.66	0.66	0.66	0.64
	Lancet^1^	0.58	0.58	0.61	0.61	0.62	0.62	0.62	0.64	0.63
	BCR^1^	0.54	0.57	0.54	0.56	0.55	0.56	0.59	0.60	0.63

Lancet	BCR^2^	0.55	0.59	0.57	0.55	0.56	0.55	0.54	0.57	0.53
	CCR^1^	0.55	0.56	0.54	0.55	0.59	0.56	0.59	0.58	0.59
	CCR^2^	0.61	0.62	0.64	0.56	0.61	0.60	0.60	0.60	0.59
	70-Gene-Sig^1^	0.56	0.55	0.53	0.57	0.58	0.60	0.57	0.61	0.61

Nature	Nature^1^	0.68	0.65	0.65	0.65	0.72	0.70	0.72	0.70	0.66
	JNCI^1^	0.71	0.71	0.65	0.61	0.59	0.64	0.60	0.57	0.61
	JNCI^2^	0.67	0.70	0.63	0.60	0.66	0.74	0.73	0.74	0.72
